# Cuproptosis-Related lncRNAs are Biomarkers of Prognosis and Immune Microenvironment in Head and Neck Squamous Cell Carcinoma

**DOI:** 10.3389/fgene.2022.947551

**Published:** 2022-07-22

**Authors:** Liuqing Yang, Jinling Yu, Lu Tao, Handan Huang, Ying Gao, Jingjing Yao, Zhihui Liu

**Affiliations:** Department of Prosthodontics, Hospital of Stomatology, Jilin University, Changchun, China

**Keywords:** head and neck squamous cell carcinoma, immune microenvironment, tumor mutational burden, cuproptosis, lncRNA, prognostic marker, drug sensitivity

## Abstract

**Background:** Cuproptosis is a new type of cell death that induces protein toxic stress and eventually leads to cell death. Hence, regulating cuproptosis in tumor cells is a new therapeutic approach. However, studies on cuproptosis-related long noncoding RNA (lncRNA) in head and neck squamous cell carcinoma (HNSC) have not been found. This study aimed to explore the cuproptosis-related lncRNAs prognostic marker and their relationship to immune microenvironment in HNSC by using bioinformatics methods.

**Methods:** RNA sequencing, genomic mutations, and clinical data of TCGA_HNSC were downloaded from The Cancer Genome Atlas. HNSC patients were randomly assigned to either a training group or a validation cohort. The least absolute shrinkage and selection operator Cox regression and multivariate Cox regression models were used to determine the prognostic model in the training cohort, and its independent prognostic effect was further confirmed in the validation and entire cohorts.

**Results:** Based on previous literature, we collected 19 genes associated with cuproptosis. Afterward, 783 cuproptosis-related lncRNAs were obtained through coexpression. Cox model revealed and constructed eight cuproptosis-related lncRNAs prognostic marker (AL132800.1, AC090587.1, AC079160.1, AC011462.4, AL157888.1, GRHL3-AS1, SNHG16, and AC021148.2). Patients were divided into high- and low-risk groups based on the median risk score. The Kaplan–Meier survival curve revealed that the overall survival between the high- and low-risk groups was statistically significant. The receiver operating characteristic curve and principal component analysis demonstrated the accurate prognostic ability of the model. Univariate and multivariate Cox regression analysis showed that risk score was an independent prognostic factor. In addition, we used multivariate Cox regression to establish a nomogram of the predictive power of prognostic markers. The tumor mutation burden showed significant differences between the high- and low-risk groups. HNSC patients in the high-risk group responded better to immunotherapy than those in the low-risk group. We also found that risk scores were significantly associated with drug sensitivity in HNSC.

**Conclusion:** In summary, our study identified eight cuprotosis-related lncRNAs signature of HNSC as the prognostic predictor, which may be promising biomarkers for predicting the benefit of HNSC immunotherapy as well as drug sensitivity.

## Introduction

Head and neck squamous cell carcinoma (HNSC) is a malignant tumor that affects different tissues and organs of the head and neck and poses a serious threat to human health (Siegel et al., 2018). HNSC is a complex disease. Typical risk factors are smoking, excessive alcohol consumption, and human papillomavirus (Jamal et al., 2016; Rooper et al., 2020). Although the current treatment methods for HNSC include surgery and chemoradiotherapy, the recurrence rate and metastasis risk of HNSC are still very high (Camisasca et al., 2011). In addition, the 5-year survival rate is less than 50% (Amit et al., 2013). An emerging approach to the treatment of HNSC is urgently needed. Hence, it is of great clinical significance to identify reliable biomarkers for predicting treatment response and prognosis and to develop effective treatment strategies for HNSC patients.

Cuproptosis is a new type of cell death that is different from the known cell death mechanism such as apoptosis, autophagy, and ferroptosis. When the known cell death mechanism is blocked, copper ions can still induce cell death. Cuproptosis occurs through the direct binding of copper ions to lipoacylated components of the tricarboxylic acid cycle in mitochondrial respiration, resulting in the aggregation of lipoacylated proteins. In addition, copper ions can reduce the protein level of the Fe–S cluster. They both induce protein toxic stress response and eventually lead to death (Tsvetkov et al., 2022). Based on this novel approach to cell death, we are developing new therapies for HNSC patients. Therefore, identifying the key regulators of cuproptosis is an important step toward further understanding.

Long noncoding RNA (lncRNA) are single-stranded RNAs with over 200 nucleotides in length, most of which do not have protein-coding capabilities (Gao et al., 2020). LncRNA regulates a variety of physiological and biochemical cellular processes by mediating chromosomal modification, transcriptional activation, and interference (Statello et al., 2021). Studies have shown that lncRNA is abnormally expressed and regulated in a variety of tumors (Castro-Oropeza et al., 2018). It has been reported that abnormal lncRNAs can be used as prognostic indicators of various cancers (Ai et al., 2020; Gai et al., 2020; Jiang et al., 2021). At present, there are few studies on cuproptosis-related lncRNAs and their association with the prognosis of HNSC patients. Therefore, this study aims to explore prognostic cuproptosis-related lncRNAs markers, to improve current strategies for diagnosis, treatment, follow-up, and prevention of HNSC.

In this study, we obtained HNSC RNA sequencing (RNA-seq) data downloaded from The Cancer Genome Atlas (TCGA) database and randomly assigned patients to a training and test datasets. We identified the cuproptosis-related lncRNAs prognostic marker (CRLPM) and developed an lncRNA signature prognostic model, which might represent potential therapeutic targets and provide valuable clinical utility for prognostic prediction of patients with HNSC. Last, we verified the predictive capacity of the model in order to provide a basis for the development of appropriate clinical strategies and revealed its potential to predict immunotherapy and drug sensitivity of HNSC.

## Materials and Methods

### Download and Processing of Transcriptomic Data, Mutation Data, and Clinical Information

The RNA-seq transcriptome profiling dataset comprised 44 normal tissues and 504 HNSC samples, which were downloaded from TCGA (https://portal.gdc.cancer.gov/) database on April 20, 2022. The tumor somatic mutation data and clinical information including survival time, survival status, age, gender, grade, stage, and tumor-node-metastasis classification were also obtained from TCGA. The annotations for lncRNAs were obtained from the GENCODE website (https://www.gencodegenes.org/). Furthermore, cuproptosis-related genes were obtained based on previous literature (Tsvetkov et al., 2022).

### Generation and Assessment of the Cuproptosis-Related Long Noncoding RNA

The coexpression analysis between cuproptosis-related genes and lncRNAs was performed by the “limma” package in R to obtain the cuproptosis-related lncRNAs. Meeting the |Cor|>0.4 and *p* < 0.001 criteria indicated an association. According to the results of the coexpression analysis, we used R “ggplot2,” “ggalluvial,” and “dply” packages to generate the Sankey plot.

### Prognostic Model Construction

The samples were randomly divided into training and validation groups through the R package “caret.” Univariate Cox proportional risk regression was performed for each cuproptosis-related lncRNAs with survival data using the survival R package. We performed the least absolute shrinkage and selection operator (Lasso)-penalized Cox regression by using the “glmnet” package in R software to avoid overfitting. The optimal and minimum criteria for the penalty (λ) using 10 times cross-validation were selected. Next, multiple stepwise COX regression analyses were performed to identify the CRLPM. Afterward, the formula of the risk scoring model was established as follows: $\text{Lasso Risk Score}=\sum_{i=1}^{n}Coef_{i}^{*}x_{i}$ , where $Coef_{i}$ represents the coefficients and $x_{i}$ represents the normalized count of each cuproptosis-related lncRNAs. Based on the Lasso prognostic model, patients can get a risk score.

### Validation of Risk Models

Patients in the training and validation groups were categorized into the high- and low-risk groups based on the median risk score and the corresponding coefficient of the training group. The Kaplan–Meier method was then conducted to display the prognostic performance of the risk score model in both the training and validation groups. In addition, the receiver operating characteristic (ROC) curve and the area under the curve (AUC) were used to evaluate the accuracy and diagnostic value of the CRLPM through the use of the survival ROC and time ROC packages in R. The principal component analysis (PCA) was also conducted to validate risk models, and the results were visualized using “scatterplot3D” packages in R software. The progression-free survival (PFS) was performed through “survival” and “survminer” packages in R. We used the C-index to predict the accuracy of risk models by using the R package “rms,” “dplyr,” “survival,” and “pec.” The validation and entire cohorts were performed to validate this model.

### Establish and Evaluate a Nomogram

We used univariate and multivariate Cox regression to investigate the independent prognostic role of the risk model. Based on the results of univariate and multivariate COX regression, we developed a nomogram by employing the R package “rms,” “regplot,” and “survival.” The accuracy of the nomogram was evaluated using a calibration curve.

### Exploration of the Relationship Between the Prognostic Risk Score and Clinical Stage

To verify whether the model is suitable for patients with different clinical stages, we explore the relationships between risk score and clinical stage to reveal their possible roles in HNSC using univariate and multivariate Cox regression analyses.

### Pathway Enrichment Analysis and Gene set Enrichment Analysis

The differentially expressed genes (DEGs) between the high- and low-risk groups were identified using the R package “limma,” with the limited condition set to log_2_ |fold change| >1 and false discovery rate < 0.05. Based on the R package “clusterProfiler,” “org.Hs.eg.db,” and “enrichplot,” we explored the Gene Ontology (GO) and Kyoto Encyclopedia of Genes and Genomes (KEGG) database pathways to clarify the molecular functions and key signaling pathways.

### Estimation of Intratumoural Immune Cell Infiltration and Immunotherapy

To investigate the relationship between the CRLPM risk score and immune cell infiltration, the single-sample gene set enrichment analysis (ssGSEA) algorithms function package in the R software genome variation analysis package was used to evaluate the infiltration and function of tumor-infiltrating immune cells. The related heat map was utilized to drawn. Next, based on the simulation of the tumor immune escape mechanism, the tumor immune dysfunction and exclusion (TIDE) algorithm was applied to predict the response to immunotherapy (http://tide.dfci.harvard.edu) (Jiang et al., 2018). Therefore, we observed the effect of immunotherapy in the high- and low-risk groups based on the TIDE algorithm.

### Evaluation of Drug Sensitivity

IC50 represented the semiinhibitory concentration of the measured antagonist. To evaluate CRLPM in the clinic for HNSC treatment, we calculated the IC50 of the chemotherapeutic drugs through the “pRRophetic” R package and its dependencies including “car, ridge preprocessCore, genefilter and sva.” A total of 138 drugs were included such as midostaurin, temsirolimus, tipifarnib, and imatinib. The Wilcoxon sign rank test was used to compare IC50 differences between common antineoplastic agents in the high- and low-risk groups. The boxplot was presented using the R package “ggplot2.”

### Calculation of Tumor Mutation Burden Scores

Tumor mutational burden (TMB) reflects the number of mutations in cancer mutation. The mutation data of HNSC samples downloaded from TCGA were analyzed using the R package “maftools.” The waterfall diagram showed the relationship between risk scores and TMB in HNSC patients.

### Statistical Analysis

All statistical analyses were processed by the R programming language (Version 4.0.3) on R studio. RNA-seq transcriptome data and somatic mutation data downloaded from TCGA were combined using the “limma” package in R. Pearson correlation test was used to analyze the correlations between cuproptosis-related genes and cuproptosis-related lncRNAs. CRLPM were screened for differential genes using the “limma” R package. Cox regression and survival analysis were performed through “survival” and “survminer” packages in R. Cox proportional risk regression model was used to calculate the hazard ratios of univariate and multivariate analyses. The GO terms and KEGG pathways were analyzed by using “clusterProfiler” in the R package. The “Pheatmap” R package was used to draw heat maps in cluster analysis. We applied the Wilcoxon rank-sum test to compare the difference between two groups of quantitative data. The overall survival (OS) time of the different groups was evaluated using the Kaplan–Meier analysis with a log-rank test. The chi-square test was used to compare categorical data between different groups. A *p* value of <0.05 was considered statistically significant.

## Results

### Data Processing

**FIGURE 1:**
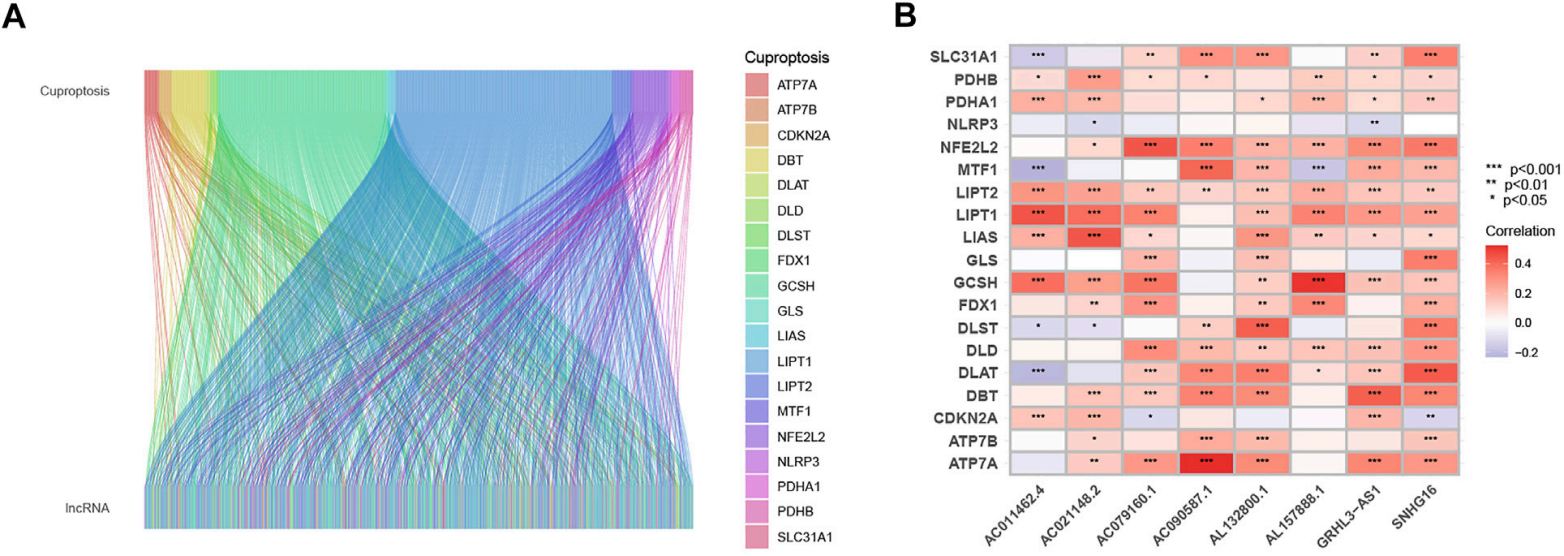
Sankey diagram and heat map. **(A)** Sankey diagram of coexpression between 19 cuproptosis-related genes and 783 cuproptosis-related long noncoding RNA (lncRNAs). **(B)** correlation 19 cuproptosis-related genes and 8 prognostic cuproptosis-related lncRNAs. *p < 0.05, **p < 0.01, and ***p < 0.001.

We removed the genes encoding proteins and identified 16876 lncRNAs in the TCGA_HNSC dataset through the “GENCODE” database. In total, we collected 19 cuproptosis-related genes. Based on Pearson analysis, 783 cuprotosis-related lncRNAs were obtained. The Sankey plot showed the association between cuproptosis-related genes and cuproptosis-related lncRNAs (Figure 1A). Thenceforth, univariate COX regression analysis was applied to explore cuproptosis-related lncRNAs (*p* < 0.05). The 501 patients were divided into the training group (*n* = 251) and the validation group (*n* = 250), and the clinical information on HNSC was presented in Table 1. The results showed that there was no difference between the training and validation groups in all clinical traits.

**TABLE 1:** Characteristic of head and neck squamous cell carcinoma patients.

Variable	Entire cohort (*n* = 501)	Validation cohort (*n* = 250)	Training cohort (*n* = 251)	*p* value
Age				
≤65	326 (65.07%)	170 (68%)	156 (62.15%)	0.2008
>65	175 (34.93%)	80 (32%)	95 (37.85%)	—
Gender				
FEMALE	133 (26.55%)	59 (23.6%)	74 (29.48%)	0.1647
MALE	368 (73.45%)	191 (76.4%)	177 (70.52%)	—
Grade				
G1	61 (12.18%)	29 (11.6%)	32 (12.75%)	0.9801
G2	299 (59.68%)	151 (60.4%)	148 (58.96%)	—
G3	119 (23.75%)	59 (23.6%)	60 (23.9%)	—
G4	2 (0.4%)	1 (0.4%)	1 (0.4%)	—
Unknow	20 (3.99%)	10 (4%)	10 (3.98%)	—
Stage				
Stage I	25 (4.99%)	10 (4%)	15 (5.98%)	0.4337
Stage II	69 (13.77%)	33 (13.2%)	36 (14.34%)	—
Stage III	79 (15.77%)	44 (17.6%)	35 (13.94%)	—
Stage IV	260 (51.9%)	121 (48.4%)	139 (55.38%)	—
Unknow	68 (13.57%)	42 (16.8%)	26 (10.36%)	—
T stage				
T0	1 (0.2%)	1 (0.4%)	0 (0%)	0.3265
T1	45 (8.98%)	19 (7.6%)	26 (10.36%)	—
T2	133 (26.55%)	61 (24.4%)	72 (28.69%)	—
T3	96 (19.16%)	54 (21.6%)	42 (16.73%)	—
T4	171 (34.13%)	80 (32%)	91 (36.25%)	—
Unknow	55 (10.98%)	35 (14%)	20 (7.97%)	—
M stage				
M0	185 (36.93%)	98 (39.2%)	87 (34.66%)	0.9569
M1	1 (0.2%)	0 (0%)	1 (0.4%)	—
Unknow	315 (62.87%)	152 (60.8%)	163 (64.94%)	—
N stage				
N0	170 (33.93%)	87 (34.8%)	83 (33.07%)	0.7488
N1	66 (13.17%)	31 (12.4%)	35 (13.94%)	—
N2	165 (32.93%)	75 (30%)	90 (35.86%)	—
N3	7 (1.4%)	3 (1.2%)	4 (1.59%)	—
Unknow	93 (18.56%)	54 (21.6%)	39 (15.54%)	—

### Construction and Validation of the Cuproptosis-Related Long Noncoding RNAs Prognostic Marker

**FIGURE 2:**
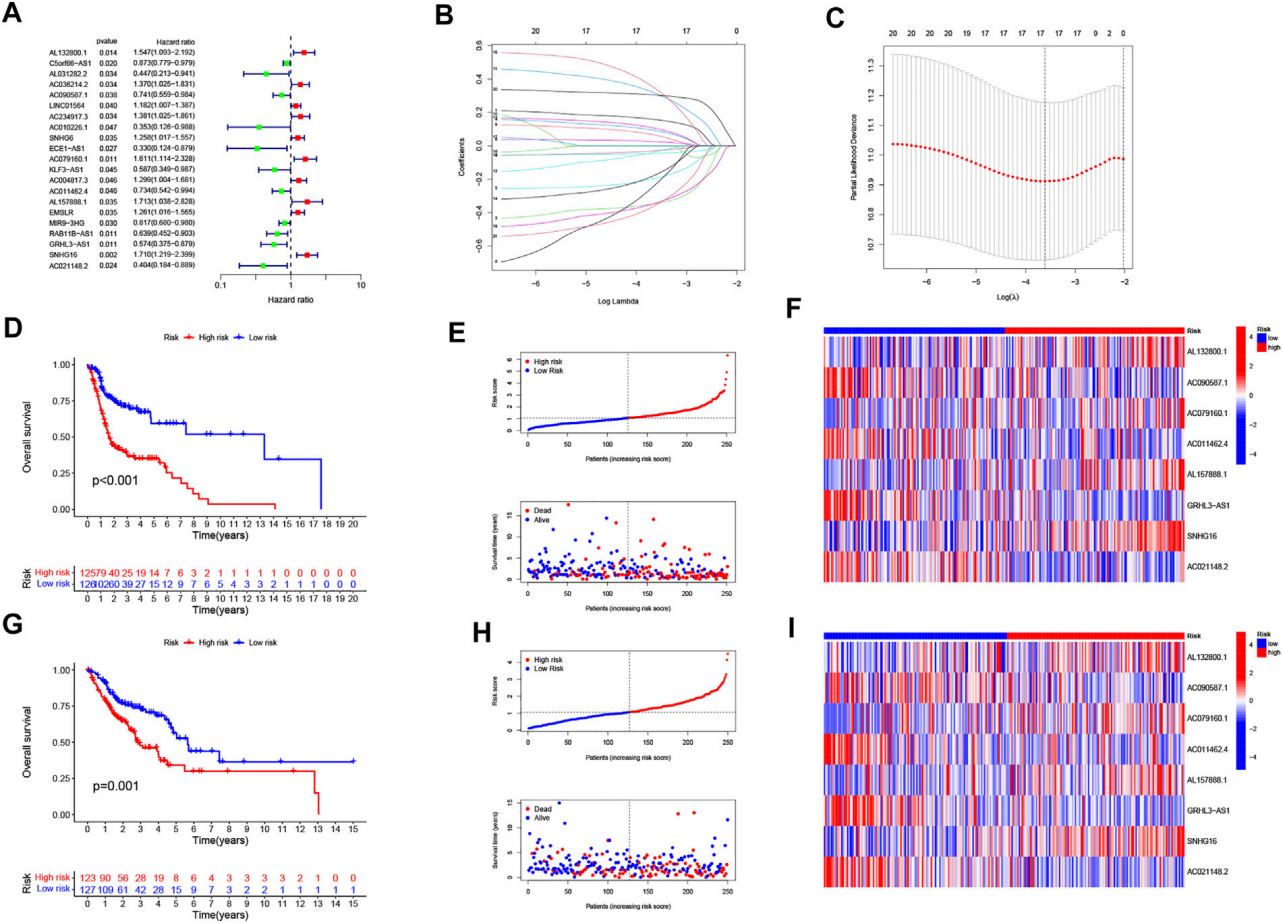
Construction of the prognostic cuproptosis-related long noncoding RNA (lncRNAs) risk model in head and neck squamous cell carcinoma (HNSC). **(A)** univariate Cox regression analysis for identifying the prognostic cuproptosis-related lncRNAs. **(B–C)** Lasso–Cox regression analysis was performed to construct prognostic prediction models. **(D)** Kaplan–Meier curves for survival analysis in the high- and low-risk groups. **(E)** risk score distribution and survival status in patients with HNSC. **(F)** heatmap of the prognostic markers and overall survival. **(G)** Kaplan–Meier curves for survival analysis in the validation cohort. **(H)** risk score distribution and survival status in the validation cohort. **(I)** heatmap of the prognostic markers and overall survival in the validation cohort.

**FIGURE 3:**
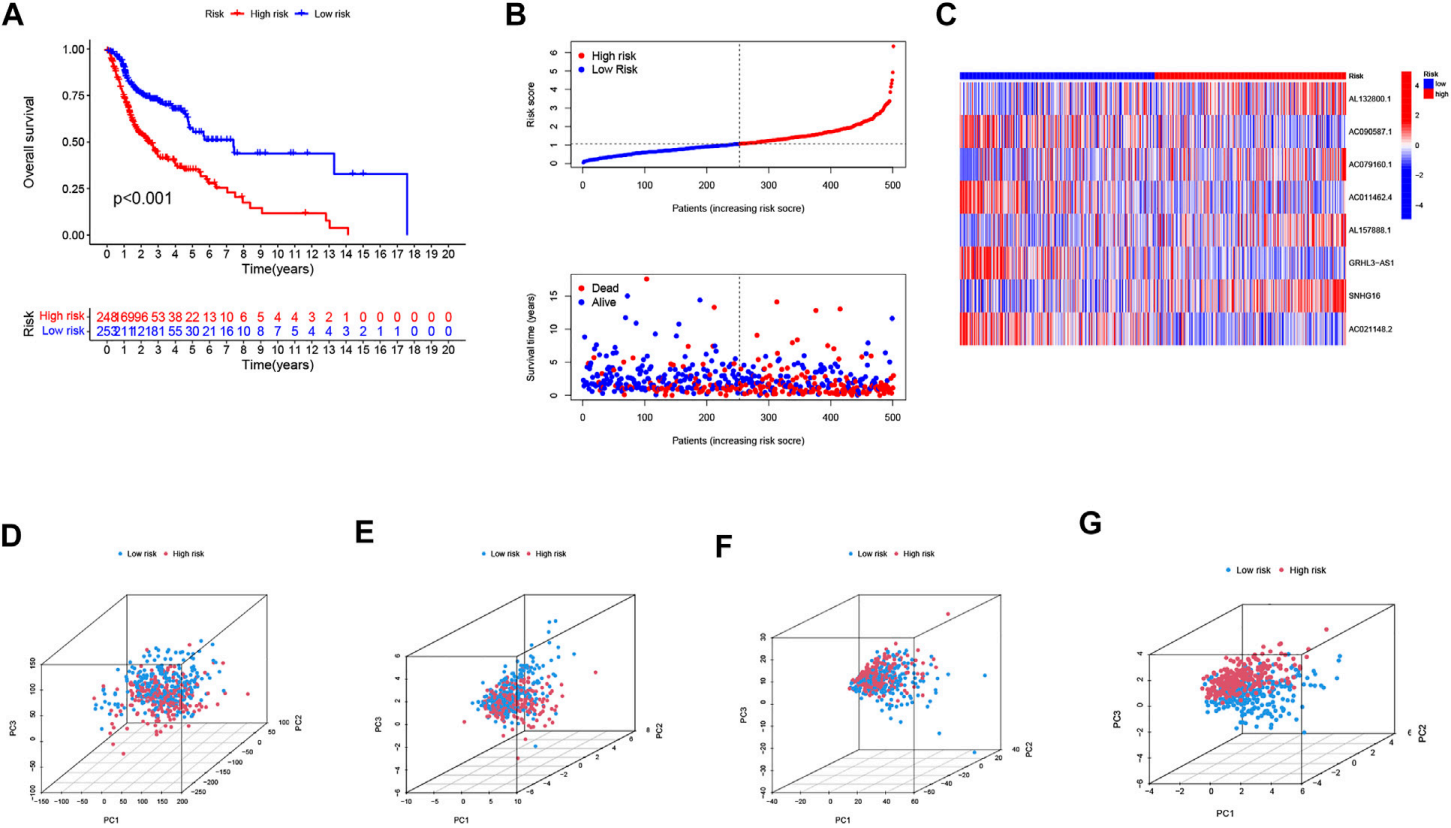
Validation of the risk model in the entire cohort and principal component analysis. **(A)** Kaplan–Meier curves for survival analysis in the entire cohort. **(B)** risk score distribution and survival status in the entire cohort. **(C)** heatmap of the prognostic markers and overall survival in the entire cohort. PCA between the high- and low-risk groups based on the **(D)** all genes, **(E)** cuproptosis-related genes, **(F)** cuproptosis-related long noncoding RNA (lncRNAs), and **(G)** cuproptosis-related lncRNAs prognostic marker.

 The univariate COX analysis of 21 cuproptosis-related lncRNAs was shown in Figure 2A. We further screened 17 lncRNAs using Lasso–Cox regression. We identified trajectory changes in regression coefficients of lncRNAs and cross-validation results of model construction (Figure 2B, C). Afterward, multiple stepwise Cox regression analysis was performed, and we screened out eight CRLPM with survival to establish the risk score models. In conclusion, we generated a total of eight CRLPMs to participate in the construction of a prognostic model to predict the OS of patients with HNSC. Afterward, we obtained a heat map of the correlation between cuproptosis-related genes and CRLPM (Figure 1B). Risk score = (0.351771323*Expression_AL132800.1_) + (−0.378321346*Expression_AC090587.1_) + (0.404882025*Expression_AC079160.1_) + (−0.314303555*Expression_AC011462.4_) + (0.716547372* Expression_AL157888.1_) + (−0.593212656*Expression_GRHL3-AS1_) + (0.390726744*Expression_SNHG16_) + (−0.892732753*Expression_AC021148.2_). Based on the median risk score, we divided the patients in the training group into the high- and low-risk groups for survival analysis. The KM method was used to analyze the OS of patients in the two groups, and the results showed that the OS of patients in the high-risk group was significantly poorer than that in the low-risk group (*p* < 0.05; Figure 2D). The distribution of risk scores and the survival status of patients were shown in Figure 2E. The expression level of eight cuproptosis-related lncRNAs involved in the high- and low-risk groups was shown in a heatmap (Figure 2F). It can be observed that with the increase in risk score, the survival time was shortened and the number of deaths increased. We also observed statistically significant differences in OS between the high- and low-risk groups in the validation and entire cohorts (*p* < 0.05; Figure 2G–I, Figure 3A–C). The PCA revealed a high degree of differentiation between the high- and low-risk groups. Based on the risk model of cuproptosis-related lncRNAs, we intuitively observed that HNSC patients were effectively divided into two clusters (Figure 3D–G).

### Independence of the Cuproptosis-related lncRNAs Prognostic Marker in Predicting Overall Survival

**FIGURE 4:**
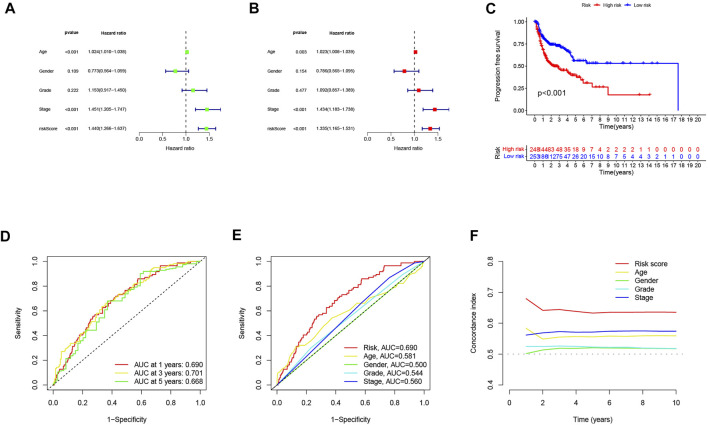
Independent prognostic analysis of head and neck squamous cell carcinoma (HNSC) overall survival (OS). **(A)** univariate Cox analysis. Age, stage, and risk score were statistically significant. **(B)** multivariate Cox analysis. Age, stage, and risk score were statistically significant. **(C)** Kaplan–Meier curves of progression-free survival (PFS). **(D)** TimeROC curve predicted 1, 3, and 5 years of OS for HNSC patients. **(E)** ROC demonstrated the predictive accuracy of the risk model was superior to other clinical parameters. **(F)** C-index showed the predictive accuracy of the risk model was superior to other clinical parameters.

**FIGURE 5:**
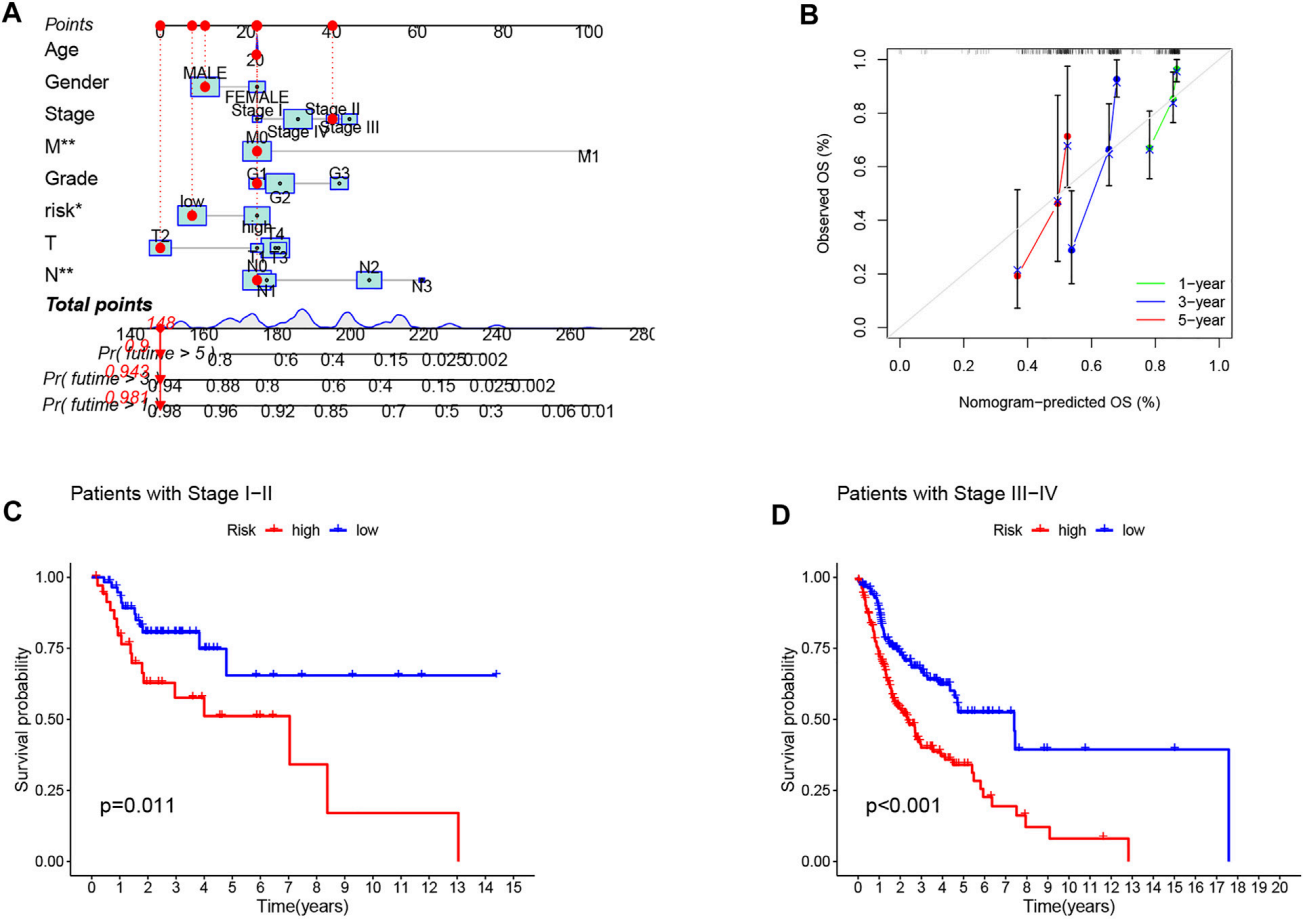
Construction and evaluation of a nomogram based on CRLPM. **(A)** nomogram used to predict prognosis was constructed based on CRLPM. **(B)** calibration curves are used to predict 1-, 3-, and 5-year overall survival. **(C)** Kaplan–Meier curves of patients with stage I-II. **(D)** Kaplan–Meier curves of patients with stage III–IV.

Univariate and multivariate Cox regression analyses were performed to assess the predictive value of the prognostic model. In univariate Cox analysis, there were statistically significant differences among age, stage, and risk score (Figure 4A). In multivariate Cox regression analysis, they remained prognostic value for OS (Figure 4B). The PFS indicated significant differences in progression-free survival between the high- and low-risk groups (*p* < 0.05, Figure 4C). The ROC curves demonstrated the accuracy and diagnostic value of the cuproptosis-related lncRNAs for OS, and the AUC reached 0.690 at 1 year, 0.701 at 2 years, and 0.668 at 3 years (Figure 4D). Both C-index and ROC curve indicated the predictive accuracy of the prognostic model was superior to other clinical including age, gender, grade, and stage (Figures 4E, F). A nomogram plot is a predictive tool for quantitative analysis of clinical outcomes in patients with HNSC. Thus, we initiated a prognostic nomogram based on the risk score and other clinical characteristics (Figure 5A). The calibration plots showed good conformity with the prediction of this nomogram (Figure 5B).

### Relationship Between the Marker and the Clinical Features in Head and Neck Squamous Cell Carcinoma

Next, to investigate the clinical utility of the CRLPM, we explored the relationship of the CRLPM with clinical features. The results indicated that there were significant differences between the distribution of risk scores and clinical stages. In specific, stages I–II and III–IV were statistically significant (*p* < 0.05, Figure 5C, D).

### Pathway Enrichment Analysis and Gene Set Enrichment Analysis

**FIGURE 6:**
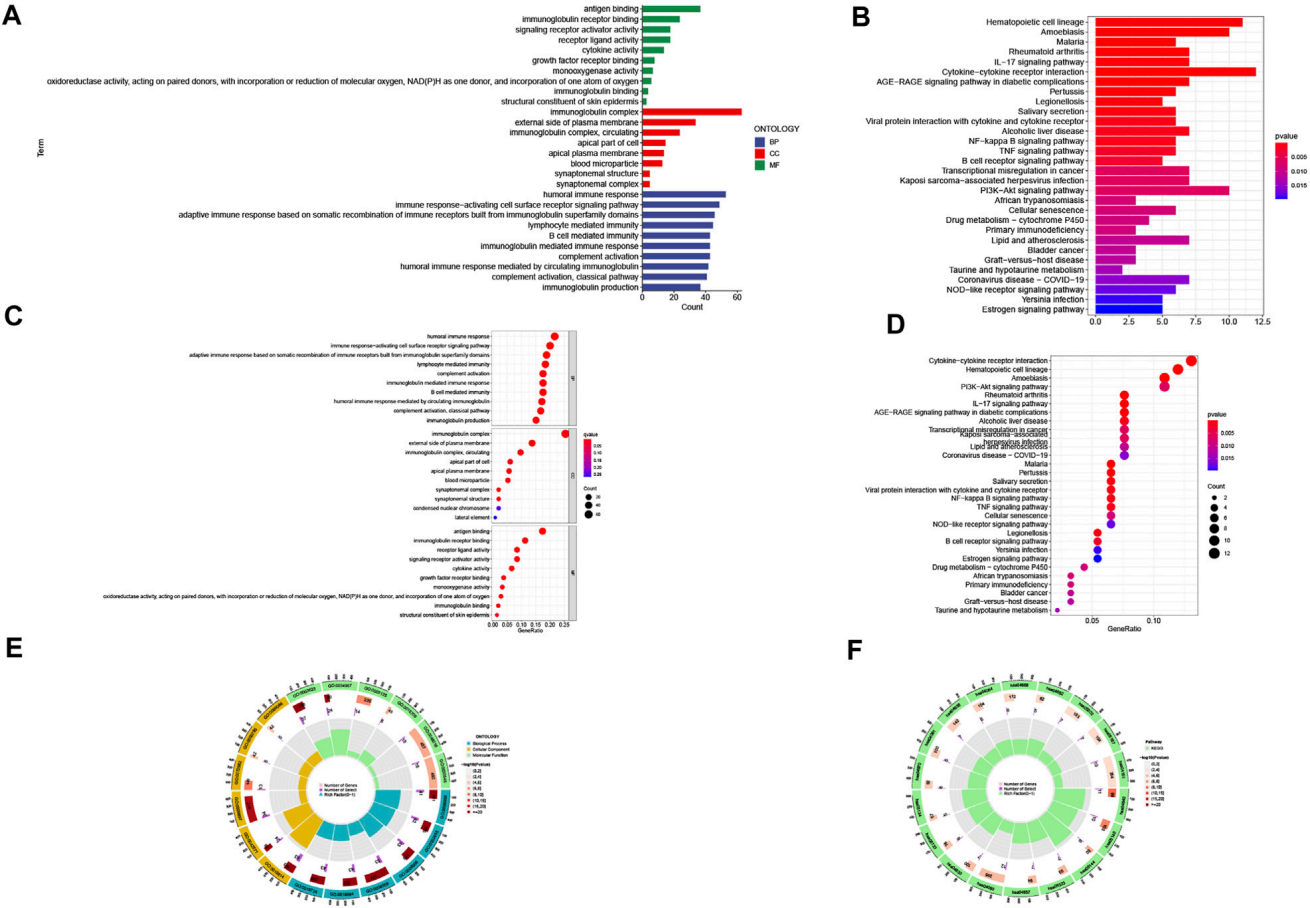
Gene Ontology (GO) and Kyoto Encyclopedia of Genes and Genomes (KEGG) pathway enrichment analysis. **(A)** barplot of the top 10 GO enrichment terms. **(B)** barplot of the top 30 KEGG enrichment terms. **(C)** bubble chart of the top 10 GO enrichment terms. **(D)** bubble chart of the top 30 KEGG enrichment terms. **(E)** circle diagram of GO enrichment analysis. **(F)** circle diagram of KEGG enrichment analysis. Biological process, cellular component, and molecular function.

To explore the biological functions and pathway analysis of DEGs between the high- and low-risk groups, we further performed the GO and KEGG enrichment analyses. A total of 359 DEGs were identified. In the biological process category, the genes were primarily concentrated in response to humoral immune response, immune response-activating cell surface receptor signaling pathway, and adaptive immune response based on somatic recombination of immune receptors built from immunoglobulin superfamily domains. In the cellular component category, it was mainly enriched in the immunoglobulin complex, external side of plasma membrane and apical part of cell. In the molecule function category, it was antigen binding, immunoglobulin receptor binding, and receptor ligand activity (Figure 6A, C, E). Genes in the KEGG category were enriched in the IL-17 signaling pathway, hematopoietic cell lineage, and amoebiasis (Figure 6B, D, F).

### Estimation of Intratumoural Immune Cell Infiltration and Immunotherapy

**FIGURE 7:**
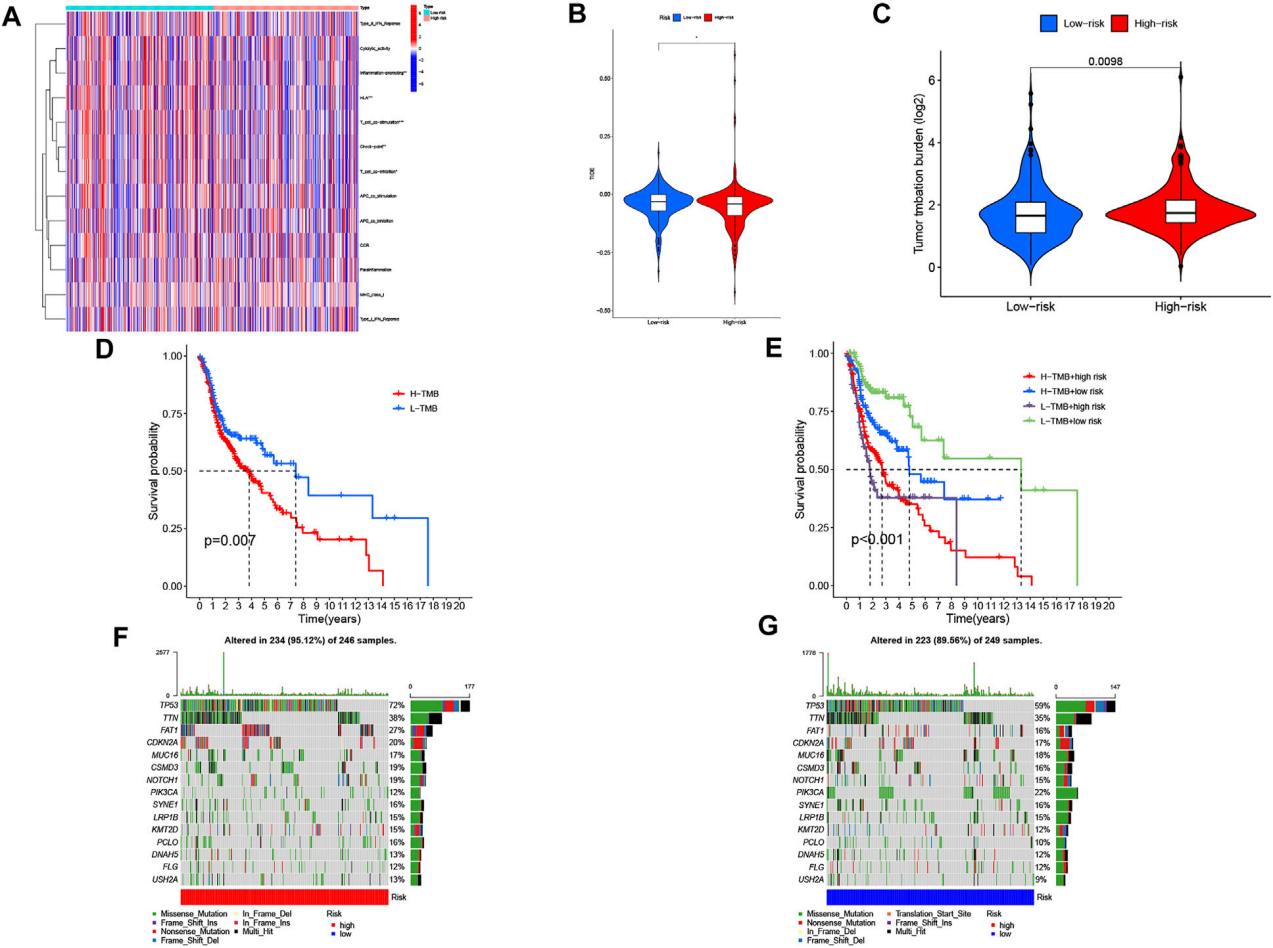
Immunological landscape in head and neck squamous cell carcinoma (HNSC) patients and relationship between tumor mutation burden (TMB) and risk score. **(A)** heatmap of the tumor-infiltrating lymphocytes based on single-sample gene set enrichment analysis algorithms among the high- and low-risk groups in HNSC. *p < 0.05, **p < 0.01, and ***p < 0.001. **(B)** comparison of TIDE prediction score between the high- and low-risk groups. **(C)** Analysis of TMB differences between the high- and low-risk groups in HNSC. **(D)** survival analysis curves of the high- and low-TMB groups. **(E)** TMB risk combined with survival curve in HNSC. **(F)** waterfall plot of top 15 mutant genes in the high-risk group in HNSC. **(G)** waterfall plot of top 15 mutant genes in the low-risk group in HNSC.

Figure 7A shows the heatmap of immune response based on the ssGSEA algorithm. Based on ssGSEA of TCGA-HNSC data, correlation analysis between immune cell populations and related functions revealed that T cell functions including regulation of inflammation, HLA, checkpoint (inhibition), and costimulation and coinhibition were significantly different between the high- and low-risk groups. These results indicated that GRLPM was associated with immune cell infiltration in HNSC. Based on the TIDE algorithm, we predicted the effect of patients receiving immunotherapy. Figure 7B shows significant differences in TIDE scores between the high- and low-risk groups and the lower TIDE scores in the high-risk group. This further proves that patients in the high-risk group have a low potential for immune escape and may receive better results from immunotherapy.

### Tumor Mutational Burden of the Cuproptosis-related lncRNAs Prognostic Marker in Head and Neck Squamous Cell Carcinoma Samples

We collected data on somatic mutation in HNSC and calculated corresponding TMB scores in order to investigate the potential role of tumor mutation load in HNSC. As shown in Figure 7C, the high-risk group had a higher mutation load than the low-risk group in HNSC. We divided patients into “Hight-TMB” and “Low-TMB” by median cutoff points and performed survival analyses. The results showed that the high-risk group had a lower survival rate than the low-risk group in HNSC (Figure 7D). A combined survival analysis of tumor mutation load and risk scores can obtain the combined survival curve. It revealed that the TMB and risk scores had significant effects on the OS of HNSC patients (Figure 7E). The mutation landscapes in the CRLPM high- and low-risk groups were compared. Waterfall plots visualized the 15 genes with the highest mutation frequency in the high- and low-risk groups. The results showed that more mutation events occurred in the high-risk group (Figures 7F, G). TP53 was the gene with the highest mutation frequency.

### Drug Sensitivity

**FIGURE 8:**
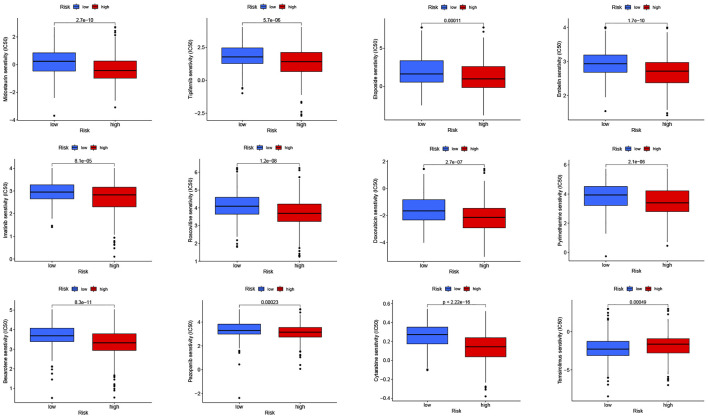
Drug sensitivity (IC50) correlated with high- and low-risk patients in head and neck squamous cell carcinoma.

To explore the possible use of CRLPM in the individualized treatment of HNSC, we investigated the relationship between risk scores and IC50 of drugs in HNSC treatment. To this end, we compared the sensitivity of 30 common anticancer drugs between the high- and low-risk groups. As shown in Figure 8, the sensitivity of 12 of the 30 anticancer drugs was significantly different in the high- and low-risk groups (*p* <0.05). Meanwhile, 11 of the drugs had lower IC50s in the high-risk group, further proving that the high-risk group was more sensitive to drug treatment. This means that these drugs have a potential role in the treatment of HNSC in the future. However, the IC50s of temsirolimus were higher in the high-risk group, which suggests that the low-risk group had a high sensitivity to this drug. Results showed that in HNSC patients, except for temsirolimus, the risk score was inversely associated with IC50 (Supplementary Figure S1).

## Discussion

Despite advances in surgery and chemotherapy in recent years, the prognosis for patients with advanced and metastatic HNSC remains poor (Miyauchi et al., 2019; Johnson et al., 2020). Cuproptosis overcomes the resistance of malignant cells to chemotherapy and helps remove defective cells. Therefore, cuproptosis may be an effective way to treat many types of cancer in the future. In addition, lncRNA affects the development and treatment of cancer through biological means (Gao et al., 2021; Tan et al., 2021). LncRNAs have been found to play an important role in the prognosis of HNSC and may be a potential effective molecular target for the treatment of HNSC (Ban et al., 2020; Tang et al., 2021; Zhu et al., 2021). However, the regulatory mechanisms of cuproptosis remain largely unknown, especially in the field of lncRNA. Therefore, we should focus on the potential interaction between lncRNA and cuproptosis to uncover potential prognostic markers.

In the current study, we identified 21 prognostic cuproptosis-related lncRNAs, eight of which were selected for prognostic construction to predict OS in patients with HNSC. First, 19 cuproptosis-related genes and 783 cuproptosis-related lncRNAs were obtained. Then, we used Lasso regression and COX regression to identify the prognostic cuproptosis-related lncRNAs. In addition, we further explored the CRLPM and common clinical variables, upstream regulatory mechanisms, immune cell infiltration and immunotherapy, and drug sensitivity of HNSC.

Based on Cox, Lasso, and multivariate Cox regression analyses, we identified eight lncRNAs associated with prognosis, including AL132800.1, AC090587.1, AC079160.1, AC011462.4, AL157888.1, GRHL3-AS1, SNHG16, and AC021148.2. Guo et al. (2021) found that AC079160.1 is a prognostic biomarker for gastric cancer, and AC079160.1 was found to be overexpressed. In general, high expression of AC079160.1 was associated with better survival in gastric cancer. AC011462.4 has been reported to play an important oncogenic role in tumors. Li et al. (2021) revealed that the high expression level of AC011462.4 was associated with longer OS. The expression level of AC011462.4 increased with the increase of risk score in colon cancer. GRHL3-AS1 expression was upregulated in patients with primary HNSC, and its expression was associated with the survival of patients with HNSC (Feng et al., 2021). Small nucleolar RNA host gene 16 (SNHG16) is considered to be a cancer-associated lncRNA that promotes tumor development primarily by acting as a competing endogenous RNA (ceRNA) (Grüll and Massé, 2019). There is evidence that SNHG16 acts as a ceRNA in various cancer by sponging corresponding miRNA to regulate mRNA (Zhao et al., 2018). In addition to the ceRNA mechanism, SNHG16 plays a role in promoting cancer through other mechanisms. SNHG16 can promote the proliferation and inhibit apoptosis of bladder cancer by inhibiting the expression of P21 (Cao et al., 2018). SNHG16 was found to be significantly upregulated in a variety of tumor tissues and cell lines, such as hepatocellular carcinoma, lung cancer, colorectal cancer, glioma, and other tumor types (Christensen et al., 2016; Yang et al., 2018; Chen et al., 2019; Su et al., 2019). In addition, high SNHG16 expression was associated with a poor prognosis. One study showed that in oral squamous cell carcinoma, the expression of SNHG16 was upregulated by c-Myc (Li et al., 2019). However, there are few reports on AL132800.1, AC090587.1, AL157888.1, and AC021148.2. Thus, it is necessary to further determine their mechanisms during cuproptosis through experiments in our future studies.

Afterward, we verify the accuracy of the risk model. The Kaplan-Meier method showed that the OS of the high-risk group was lower than that of the low-risk group. The ROC curves showed that CRLPM had high accuracy in predicting 1-, 3-, and 5-year survival, and AUC were all greater than 0.65. PCA intuitively showed differences between the high- and low-risk groups. PFS, C-index combining CRLPM with clinical information, a new nomogram was created to predict prognosis, lymph node metastasis, and distant metastasis in HNSC patients.

In addition, functional enrichment analyses revealed the potential biological mechanism of the involved CRLPM. We explored the key signaling pathways of eight cuproptosis-related lncRNAs. GO and KEGG analysis indicated that this differentially expressed CRLPM was mainly enriched in the IL-17 signaling pathway, hematopoietic cell lineage, and amoebiasis.

Our results found that the TMB was statistically higher in the high-risk group than in the low-risk group, suggesting that patients at high risk of HNSC have a better response to immunotherapy. Among the first 15 mutated genes, TP53 was mutated more frequently in HNSC patients. Moreover, the drug sensitivity of these CRPLM was analyzed to guide clinical treatment. There were significant differences in IC50 between high-risk and low-risk patients for all 12 drugs.

Cuproptosis is a new form of cell death that may play an important role in future cancer treatments. On the other hand, some lncRNAs influence cancer progression and treatment in a variety of biological ways. However, there is still a lot of unexplored territory between cuproptosis and lncRNA. Overall, this study provides new insights into the tumorigenesis and progression of HNSC from the perspective of cuproptosis. Biomarkers of cuproptosis that can be used for the prognosis of HNSC were explored, which could inform the treatment of the disease. We inevitably used only the TCGA validation and entire cohorts, and additional patients could improve the reliability of the model; thus, further validation is needed through preclinical studies. In the meantime, our study needs further validation *in vivo* and *in vitro* soon.

## Conclusion

To sum up, we identified a risk model based on seven cuproptosis-related lncRNAs that accurately predicted the prognosis of HNSC. Hence, our study provided a new therapeutic strategy for individualized therapy and immunotherapy response in HNSC patients. These eight cuproptosis-related lncRNAs may be therapeutic targets for HNSC.

## Data Availability

The original contributions presented in the study are included in the article/Supplementary Material, further inquiries can be directed to the corresponding authors.

## Supplementary Material

Supplementary Figure S1 Linear correlation between IC50 and risk score.
