# Supplementary figures and images for: Cuproptosis-Related lncRNAs are Biomarkers of Prognosis and Immune Microenvironment in Head and Neck Squamous Cell Carcinoma
Source: Front Genet. 2022 Jul 22;13:947551. doi: 10.3389/fgene.2022.947551 (PMC9354258; doi:10.3389/fgene.2022.947551)

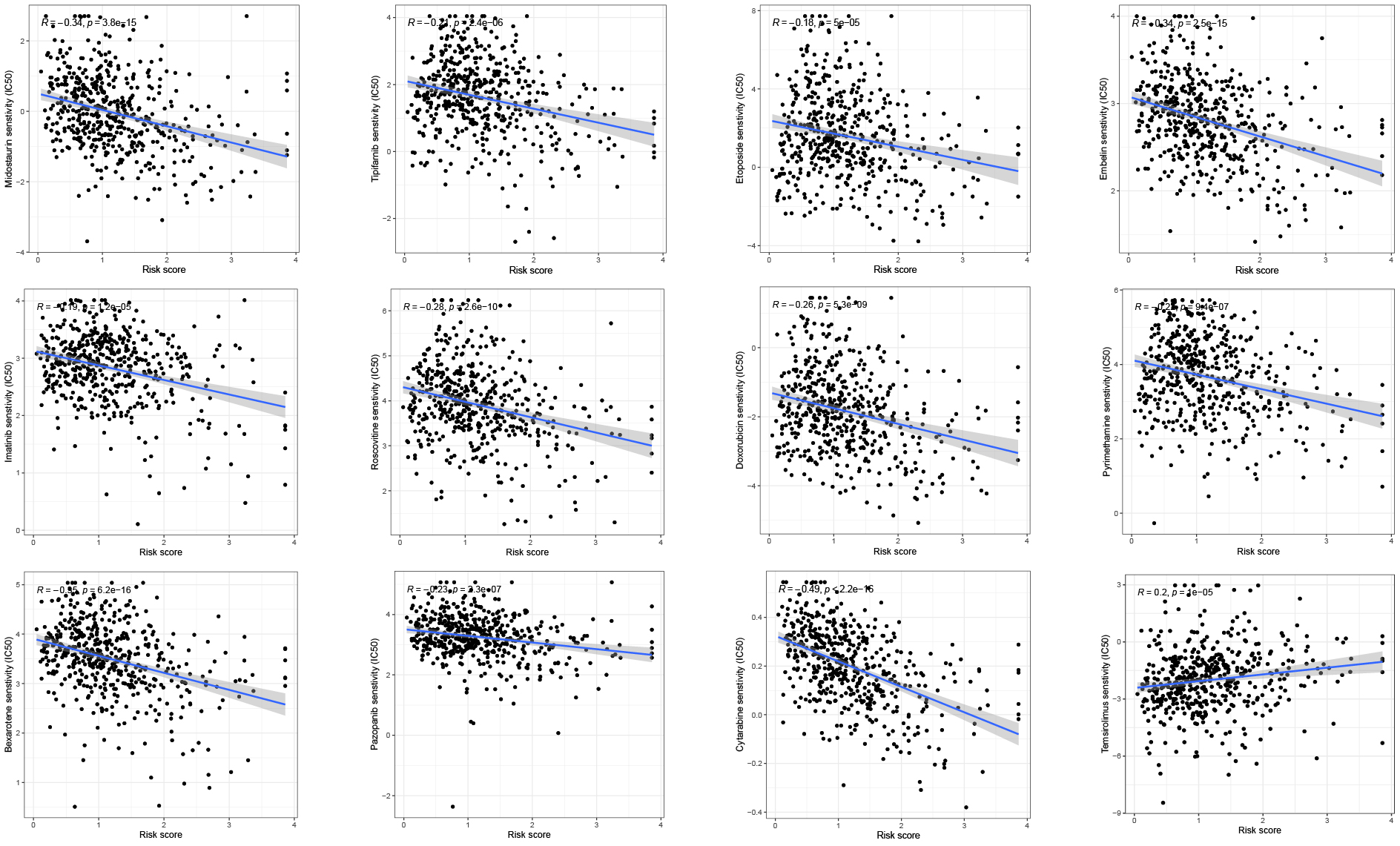

Supplement: Supplementary file 1 [file Image1.JPEG]
